# Energy as a Social and Commercial Determinant of Health: A Qualitative Study of Australian Policy

**DOI:** 10.34172/ijhpm.2022.7193

**Published:** 2022-12-07

**Authors:** Fran Baum, Michael P. McGreevy, Colin M. MacDougall, Mark Henley

**Affiliations:** ^1^Stretton Institute, University of Adelaide, Adelaide, SA, Australia.; ^2^Department of Industrial Systems Engineering and Management, National University of Singapore, Singapore, Singapore.; ^3^College of Medicine and Public Health, Flinders University, Adelaide, SA, Australia.; ^4^Uniting Care, Adelaide, SA, Australia.

**Keywords:** Energy Policy, Health, Climate Change, Equity, Australia, Commercial Determinants

## Abstract

**Background: ** This paper considers energy as a social and commercial determinant of health. Stable access to clean and sustainable energy is integral for human wellbeing yet public health rarely considers its importance.

**Methods:** Using NVivo qualitative analysis software we analysed all Australian federal, state and territory strategic energy policies covering varying periods between 2016-2030. We defined strategic policy as including the goals, objectives and strategies of the department regarding a specific area of policy responsibility. This criterion excluded documents such as operational guidelines. 36 energy-related policies were analyzed.

**Results:** While the nature of energy supply is crucial to determining the impact of human and environmental health, our analysis showed that health and wellbeing are only rarely considered in policy. We developed a conceptual framework to guide our work linking energy policy with health. Australia’s continued reliance on fossil fuels evident in the policies poses health risks, especially as climate change threatens physical and mental health. Yet health considerations were mainly absent from the policies. However, some jurisdictions (South Australia and the Australian Capital Territory [ACT]) had policies encouraging a fast move to renewables. Energy pricing was a key focus in each jurisdiction and had become highly politicalized in the past decade. Little attention was paid to equity considerations in the policies.

**Conclusion:** Energy policy would be more health promoting if public health perspectives were considered during its development. On the basis of our policy analysis and literature review we conclude with recommendations for healthy energy policy.

## Background

**Key Messages**

** Implications for policy makers**

- Australia energy policies pay little attention to the health impacts on individual and pollution health.
- Our study of Australian energy policies indicates considerable difference in the extent to which the jurisdiction embraced a transition to renewable energy away from fossil fuels.
- Governments with a commitment to healthier energy policy would ensure the policies tackle cost, increase in renewables and sustainability.
- If health and energy sectors collaborate energy policy would be more likely to be health and equity promoting and not have negative health effects.

** Implications for the public**

The policies that governments adopt in relation to energy can have a strong impact on your health and that of the whole population. A detailed analysis of energy policy in Australia shows that opportunities were lost to promote health through energy policy. Australia is still very reliant on fossil fuels which contribute to climate change. The price of energy has also increased in Australia and this has put people in to poverty and some are unable to afford sufficient heating or cooling to ensure wellbeing. Our research recommends that when energy policy is devised health considerations should inform the policy. By doing this the health of people and the planet can be enhanced.

Energy production, storage and use has a profound impact on the health of societies. Energy is both a social and commercial determinant of health. Stable access to clean and sustainable energy is integral for human wellbeing^1^ and the energy sector generates employment. Yet despite its importance the energy sector is rarely mentioned in discussions about the need for intersectoral action to tackle the underlying causes of ill health and health inequities. When it is considered^1,2^ most attention is given to the impact of using fossil fuels for energy production which produces pollution and contributes to climate change. Health and equity outcomes associated with climate change are broad and include heat stress, floods, drought, storms, increased air pollution, changed and intensified patterns of disease, food insecurity, poorer nutrition, displacement and human stress.^3^ Problems relating to fossil fuels stem from burning coal, gas and oil and associated problems from mining, transport, combustion, waste generation and disposal. The World Health Organization (WHO) point out that pollutants from energy are not only local issues but also national and global because as they can be transported via air, water and soil pollution^4^ argued that health problems from poorly managed energy generation, distribution and consumption produce large economic burdens for individuals, communities and governments – locally, nationally and globally. Energy production also makes a significant contribution to the spread of non-communicable disease, for example air pollution and water contamination are linked to heart attack, stroke, chronic obstructive pulmonary disease, cancer, respiratory infections, birth defects and asthma.^5-7^ Workplace injuries and deaths are caused by unsafe mining and transport.^1^ The growing dependence on private cars and road freight also brings health risks in the form of urban pollution, reduction in exercise and road traffic injuries. Cities have been built to accommodate private cars and the resultant urban form is characterized by sprawl, freeways and hostile environments for pedestrians and cyclists.^8,9^

 To reduce the many negative health effects from energy systems strong intersectoral coordination between health and energy sectors to produce energy policy with population health equity as a key consideration is vital. Yet WHO note that existing co-ordination is too sporadic and weak and that improved energy sector practice could reduce health service demand and expenditure.^10^ Healthy energy sectors are also vital to avoid the predictions of catastrophic global warming and subsequent climate change.^11^

 In this paper we have three aims. Firstly, we examine the extent to which Australian energy policy incorporates consideration of positive and negative health and equity outcomes from energy sources. Secondly, we synthesise findings from the policy analysis and the literature on the links between energy and health to provide a conceptual framework which lays out the pathways from energy production and use to population and individual health outcomes. Finally, we make recommendations for the ways in which the consideration of health impacts in energy policy can be improved.

###  Background to the Australian Energy System

**Figure 1:**
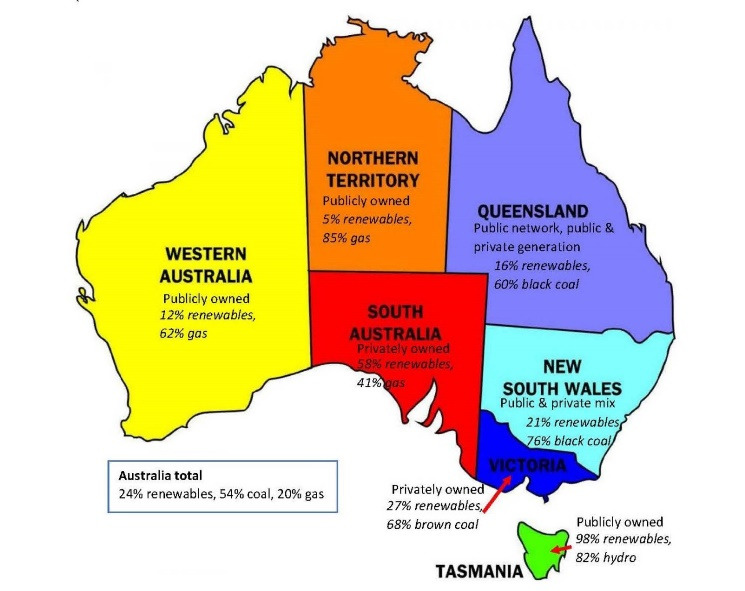
Ownership and Electricity Generation Across States (Constructed from data from^12,13^).

**Figure 2:**
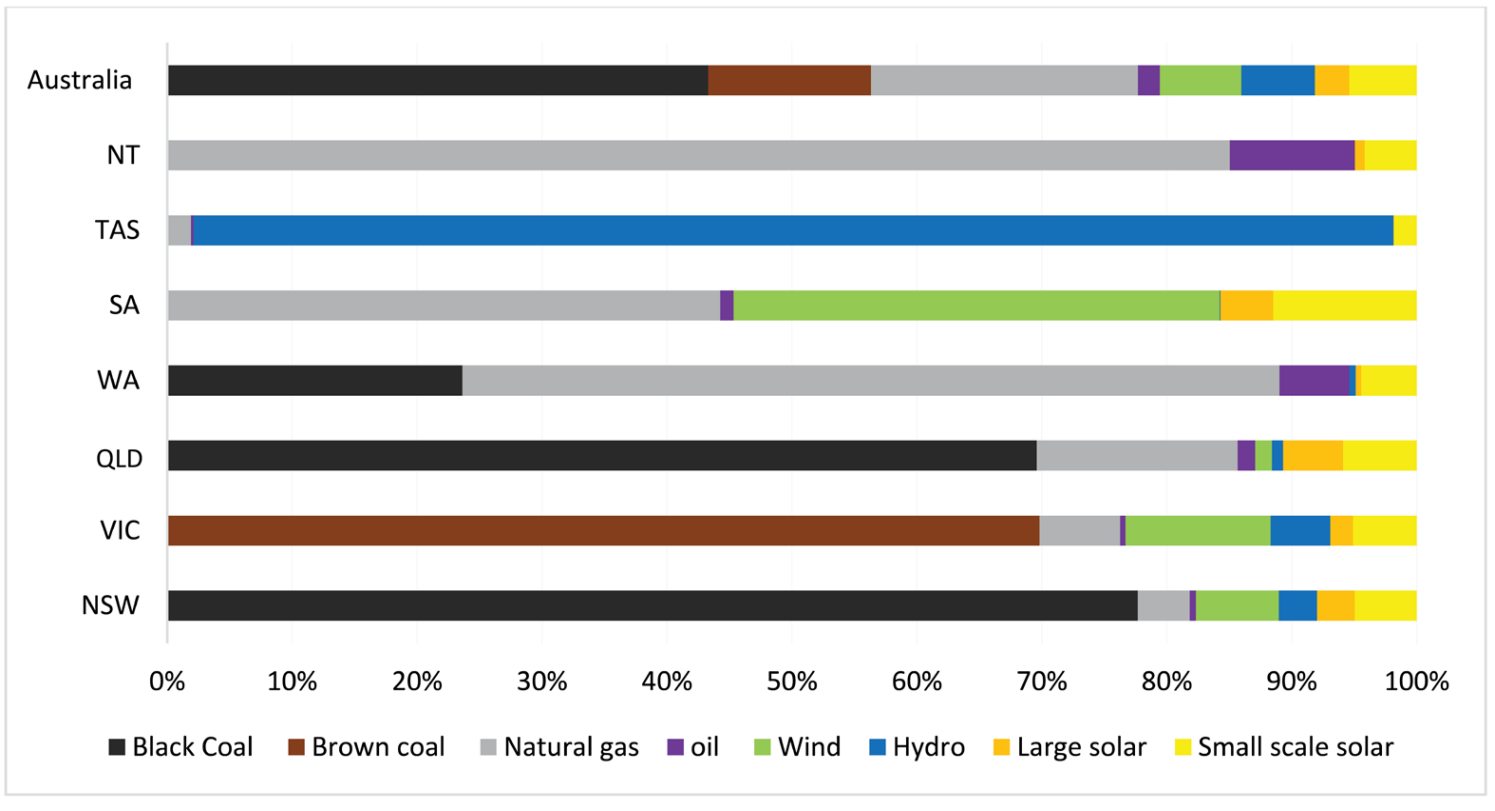
Australian Electricity Generation Resources (Constructed by authors from data^13^). Abbreviations: NT, Northern Territory; TAS, Tasmania; SA, South Australia; WA, Western Australia; QLD, Queensland; VIC, Victoria; NSW, New South Wales.

 Australia is a federated state and each jurisdiction has its own set of policies. The five eastern states and the Australian Capital Territory (ACT) are part of the National Energy Market (NEM), while Western Australia (WA) and the Northern Territory (NT) are not due to distance and isolation. WA and NT have publicly owned and run networks and retailers, and generation mostly from publicly owned plants and some private facilities including household rooftop solar. Within the NEM there is a diversity of state based public and private infrastructure providers and operators, public and private generators, and private retailers (Figure 1). Queensland’s electricity supply is in public ownership. Most significantly the nature of supply is crucial in determining the impacts on human and environmental health. Australia is still heavily dependent on electricity generation from fossil fuels, particularly coal (Figure 2). In 2018-2019, 94% of Australia’s energy use including transport and industry use was from fossil fuels. Furthermore, Australia’s total energy use has been rising at a faster rate than its uptake of renewables.^14^

 An important backdrop to our study is that in the period covered by our policy analysis energy policy was highly politicised. A carbon pricing scheme in Australia was introduced by the Gillard Labor minority government in 2011 as the Clean Energy Act 2011 which came into effect on July 1, 2012.^15,16^ Measures were also introduced to protect low-income earners from the impact of the resulting rise in energy costs. Emissions from companies subject to the scheme dropped 7% upon its introduction. This act was reversed by the incoming Abbott conservative coalition government in 2014.^15^ In the 2019 Federal election campaign carbon emissions remained a key issue and focused on whether the Adani coal mine in Queensland should go ahead.^18^ The proposal was not opposed by the two largest political parties – a conservative Liberal and National Party Coalition who were in government and the opposition Australian Labor Party – despite the likely environmental and health risks. Nevertheless, the issue was argued to have cost the Labor party vital seats in the campaign and ultimately government in 2019.^19^

 Australia is not only a major user of fossil fuels it is also a major exporter. Coal alone accounted for 15% (A$60 billion) of the country’s export income in 2018 and gas a further 8% ($38 billion).^20^ These export industries are predominantly situated in three states. Coal exports from Queensland and New South Wales (NSW) and gas from WA and Queensland. This economic pre-eminence has given the fossil fuel lobby significant political influence over some state and federal governments.^16,17,21^ Nevertheless, this influence creates a focus on the endurance of coal in a country which has considerable potential for solar and wind energy.^22^

 The cost of energy for consumers has both equity and health implications. A significant review of Australian energy markets and energy costs reported in June 2017.^23^ Central to this report was the notion of the energy ‘trilemma’ which concerned three factors: (*i*) balancing price for customers, (*ii*) ensuring reliability, and (*iii*) reducing emissions. Energy costs may preclude some people (including those with health problems and disabilities) using energy for heat or cooling. Spending on energy reduces the resources a household has for other goods that may promote health including food. Energy costs are regressive in that any increase will be proportionately a larger share of income for households with a lower income and pose an opportunity cost on lower income households, particularly renters, who are unable to afford options which would increase the thermal efficiency of their rental housing. Average household expenditure on electricity is about 2.9% of disposable income,^24^ but double that for the poorest 3 deciles of the income distribution.^25^ A further equity consideration is that concerning an eight year life expectancy difference between Aboriginal and Torres Strait Islander and other Australians.^26^ These Australians’ health may be affected through the cost of energy and mining activity on their traditional lands.

## Methods

 We used standard document analysis techniques to analyze Australian energy policies, which involved collecting, coding, synthesising and theorising the research data.^27,28^ The first step involved collecting the necessary documents.^29^ Then we reviewed each policy to determine if it was primarily a strategic document. We collected all current strategic policies, selected legislative documents and the most recent annual report from departments responsible for energy in the nine Australian jurisdictions (all state and territory governments, and the federal government) covering varying periods between 2016-2030. This resulted in 132 documents being identified. These were then examined to determine if they were strategic policy documents. We defined strategic policy as including the goals, objectives and strategies of the department regarding a specific area of policy responsibility. This criterion excluded documents such as operational policy, research papers, community consultations, budget documents, technical guides and discussion papers. We found that most of the documents were operational rather than strategic and the team collectively selected 36 energy-related policies to analyzed in detail (see Table) representing a census of all Australian strategic energy policies.

**Figure 3:**
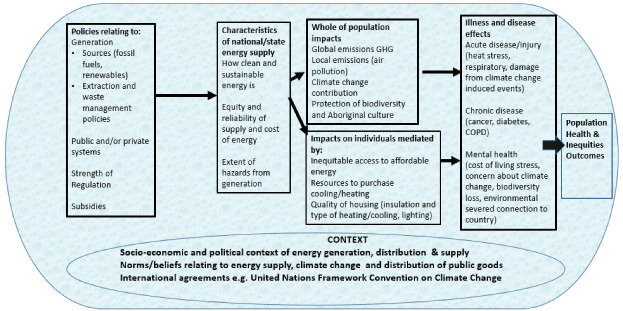
Energy as a Social and Commercial Determinants of Health: Conceptual Framework. Abbreviations: GHG, greenhouse gas; COPD, Chronic obstructive pulmonary disease.

 Our coding system was developed to capture the keywords representing the social determinants of health. The coding framework is provided in Baum et al.^30^ These codes were then used to analyse the policies to determine whether and how the policies aligned with the intent of progressing health and health equity.^31^ All documents were coded thematically using NVivo. A qualitative analysis was undertaken to review and evaluate them systematically by two researchers and two other researchers double coded a sample of the policies. Like other qualitative methods, document analysis requires data to be examined and interpreted by the researchers to elicit meaning and develop understanding about what is present and not present in the data, and to what effect.^30^ As the data analysis was conducted, we made links to the broader literature linking energy and health.^30^ The connections and resulting narrative continued during the drafting of this paper, with input from all authors. We developed a conceptual framework to guide the process of linking elements of energy policy to likely health and wellbeing outcomes (Figure 3).

**Table:** Australian Strategic Energy Policies Included for Analysis

**Jurisdiction **
**Federal Government **
Australian Renewable Energy Agency Act 2011
Australian Government & COAG Energy Council National Energy Productivity Plan: Work Plan n.d.
National Energy Productivity Plan 2015–2030
Clean Energy Legislation (Carbon Tax Repeal) Act 2014
**ACT**
Renewable Energy Industry Development Strategy
ACT Sustainable Energy Policy: Energy for a sustainable city 2011-2020
Climate Change and Greenhouse Gas Reduction Act 2010
ACT Department of Environment and Planning Corporate Plan 2015-2017
ACT Climate Change Strategy 2007-2025
ACT Climate Change Adaptation Strategy Living with a Warming Climate July 2016
**NSW**
NSW Renewable Energy Action Plan
NSW Gas Plan
Energy Efficiency Action Plan
Climate Change Fund Draft Strategic Plan 2017-2022
**NT**
Department of Mines and Energy Strategic Plan 2014-2020
Onshore Oil and Gas Guiding Principles
NT Power and Water Network Management Plan 2013-2014 to 2018-2019
**Queensland**
Strategic Plan 2016-2020 (DEWS)
A framework for the next generation of onshore oil and natural gas in Queensland
Department of Natural Resources and Mines Strategic Plan 2015-2019
Powering Queensland Plan
Queensland Renewable Energy Plan
**SA**
Low Carbon Investment Plan for SA
A Renewable Energy Plan for SA
South Australian Minerals and Energy Services Strategic Statement
Our Energy Plan 2017
**Tasmania**
Tasmania's Energy Strategy: Restoring Tasmania’s Energy Advantage
Corporate Plan 2015-2018
Tasmanian Renewable Energy Action Plan 2020
**Victoria**
Towards SV2020: Five year Strategic Plan
Corporate Plan 2015-2019 (ELWP)
DEDJTR Delivers – Strategic Plan 2016-2017
Victoria’s Renewable Energy Roadmap (DELWP)
Sustainability Victoria Business Plan 2016-2017
**WA**
Strategic Energy Initiative: Energy 2031
Our Plan for Success to 2019 (DMP)

Abbreviations: NT, Northern Territory; SA, South Australia; WA, Western Australia; NSW, New South Wales; COAG, Council of Australian Government; DEWS, Department of Energy and Water Supplies; SV, Sustainable Victoria; ELWP, Department Energy, Land, Water and Planning; DELWP, Department Energy, Land, Water and Planning; DMP, Department Mines and Planning; ACT, Australian Capital Territory; DEDJTR, Department of Economic Development, Jobs, Transport and Resources.

## Results

 During our analysis of the Australian policies, we constructed Figure 3 to display the pathways by which energy has a direct or indirect impact on health. This figure enables assessments of the ways in which energy affects health.

 Our findings discuss how direct considerations of health are framed in the policies. We then consider the extent to which policies consider climate change. Next, we consider the health impacts of the cost of energy and then the equity considerations raised by energy policy. We also consider the ways in which energy interacts with other sectors.

###  Direct Consideration of Health

 Our key finding from the policy document analysis is that health is rarely explicitly considered in Australian energy policy documents. The exceptions we found were in South Australia [SA] and the ACT. The *SA Our Energy Plan *(p. 7) noted:

 “*Local businesses and industries rely on power for their viability and householders for their daily lives, health and comfort.”*

 Explicit links to quality of life were made for instance the *ACT Sustainable Energy Policy* (2011-2020, p. 1) and these were linked to wellbeing.

 “*Energy consumption is fundamental to our quality of life. Energy powers our homes and schools, heats and cools our workplaces and hospitals and transports us in cars, buses and planes….”*

 To a lesser extent there was discussion of the environmental problem stemming from carbon emissions and their link to climate change. The Victorian Department of Economic Development, Jobs, Transport and Resources couched the move to renewable energy as one which would improve liveability and viewed energy as one part of building a “Sustainable Victoria” which aimed to offer immediate benefits and longer-term ones for future generations. Sustainable Victoria noted in its *Five-Year Strategic Plan* (p. 9) that:

 “*Energy in the form of electricity, gas, fuel and heat, provides us with the means to live comfortably in our homes, to travel to and from work, to manufacture goods and provide services, and to enjoy our leisure time.”*

 This statement indicates an appreciation of the value of energy to general societal wellbeing.

 The NT where policies were primarily technical did show some concern for health:

 “*Unmanaged air and noise emissions from oil and gas projects may present health, safety, environmental and commercial risks”* (Onshore Oil and Gas Guiding Principles, p. 10).

 Energy related legislation and policy do refer to occupational health and safety concerns with mining and production of energy. The Federal documents made very little reference to health and well-being and when they did the focus was on specific health risks. For example, the *National Energy Productivity Plan* (2015-2030, p. 21) notes in relation to fuel quality standards an ambition to “reduce the level of pollutants and emissions arising from the use of fuel that may cause environmental and health problems.”

###  Nature of Energy Market and Energy Sources and Implications for Health

 The design of a nation’s energy market affects its health impact. In 2021, the Australian electricity market is multi layered, diversely owned, complex in its structure and highly opaque in regard to its operational agreements and relationships as shown in Figure 1. Each Australian jurisdiction’s policies noted the fast-changing nature of the energy system. For instance, the electricity system in Australia has over the past 30 years transformed from a publicly owned state based vertically integrated and centrally coordinated public service to a national market with multiple actors.^32^ This is in large part driven by the 2002 Parer Review^33^ which had the policy intent of great economic efficiency and transparency resulting in better outcomes for consumers and national consistency across jurisdictions.

 Three national market bodies were established to support this approach, the Australian Energy Market Operator, the Australian Energy Market Commission, as rule maker and the Australian Energy Regulator as economic regulator and with enforcement and compliance responsibilities. These bodies focus on the economics of energy rather than consideration of health and wellbeing impacts.

 The policy analysis showed that the source of energy, and particularly the continued reliance on fossil fuels, is a crucial political issue in Australia. This was most clearly shown in the South Australian *Our Energy Plan* (2017). The Plan starts with a message from the then Premier Jay Weatherill which says (p. 1):

 “*The national energy market is failing South Australia and the nation. Our country, with its abundance of solar, wind and gas resources, is now facing an energy crisis. We also have a system that puts profits before people.”*

 He was referring to a state-wide ‘system black event’ blackout that occurred in 2016 and which the Federal government blamed on the State’s high level of renewable energy. Subsequent inquiries demonstrated this was not the case, though ‘trip setting’ that shut down wind turbines were changed, along with other technical adjustments to reduce future “system black” events. In this policy, the then Australian Labor Party government was blaming the privatisation of energy supply for the rising prices and uncertainty of supply.

 While not as critical, Queensland (a state with massive coal resources) in its Powering Queensland Plan said, in reference to the national policy, that it would continue to advocate “for stable, integrated national climate and energy policies.” In the same plan Queensland also recognised the benefits of state-ownership noting “this action is only possible because we have kept our electricity assets in public hands, enabling the Government to deliver better outcomes for Queensland electricity consumers.” The Tasmanian Strategy (p. 27) discussed the relative high use of biomass fuels and notes *“25 per cent of Tasmanian households use wood as their dominant form of heating*.” The Tasmanian Strategy does not mention the health risk of wood fire smoke, which is well recognised by the WHO.^6^

 A further point that emerged in relation to energy supply was the universal use of business language rather than that which sees energy as a public essential service. Users of energy are referred to as “customers” and energy is presented as a business. Thus, the Tasmanian Strategy had a very strong focus on restoring “energy as a competitive advantage for Tasmania” yet was silent on health and well-being considerations other than reducing the price of energy. It also talked about the need *to “reduce regulatory red tape.”*

 Energy supply was also viewed as being “uncertain” and most policies showed considerable concern with achieving a safe, secure, and reliable supply of energy – a motivation that is supportive of health. The Tasmanian Strategy (p. 9) noted:

 “*In the context of these future uncertainties and rapid changes, a particular challenge is ensuring regulatory reforms keep pace with the rapid transformations that are already being seen in the market.”*

 Finally, none of the jurisdictions promoted nuclear energy and nuclear power production is not permitted under two main pieces of Commonwealth legislation—the Australian Radiation Protection and Nuclear Safety Act 1998, and the Environment Protection and Biodiversity Conservation Act 1999. The continuation of this legislation perhaps reflects strong civil society opposition to nuclear energy in Australia and a recognition of the health harms it poses in the event of an accident of the type that occurred in Japan following the 2011 tsunami. Though the SA Government called a Royal Commission into the Nuclear Fuel cycle between March 2015 and May 2016 due to SA being actively considered as a location for a national nuclear waste dump. This idea was taken to a citizen jury which after four days of deliberation voted decisively against such a dump.^34^

###  Climate Change

 Closely related to the nature of energy supply is the consideration the polices give to climate change and its actual and projected impact on health. We found the energy policies were primarily concerned about network management, retailing, generation and specific energy sources including some focus on renewables. The main emphasis we found was on technological innovation, devising strategies to extend the economic life of non-renewable sources, and find renewable sources to ensure energy security and ongoing economic gains. The focus on fossil fuels was most evident in WA where there is a Department of Mines and Petroleum, reflecting the massive deposit of carbon-based fuels in that state. Its mission is stated in its Our Plan for Success (p. 1) as *“Encouraging responsible exploration and development of mineral and petroleum resources.”*

 Climate change is mentioned in approximately two thirds of the documents, but most often as a passing mention or in the problem framing establishing why there is a need to act, although we found little explicit discussion about the social and health harms associated with climate change and why it is in the interests of society to act. The one exception was the ACT whose Climate Change Strategy 2007-2025 which listed the health impacts of climate change as: (1) temperature-related illness and death – due to increased temperatures and heatwaves; (2) food and water-borne diseases – due to changes in water quality and the range of bacteria and pests; (3) respiratory disease – due to increased pollution; (4) mental health disorders – due to social disruptions; (5) vector-borne disease – from a change in the range of mosquitoes and other disease-carrying species; and (6) injury, trauma and related effects – from an increase in extreme weather events. There was some emphasis on renewables in all jurisdictions, but they vary in their level of support for these. Thus, the Tasmanian Plan (p. 10) stated that it *“ recognises that the world is beginning the transition to a low carbon future in response to the challenge of climate change.”* In the introduction to the Queensland Renewable Energy Plan (p. 1), the Premier was clear that her Government *“ recognises that climate change is one of the great challenges of our age.”* This document also recognised that Queensland produced more harmful greenhouse gases per person in Queensland than any other state with approximately 43 tonnes of greenhouse gas emissions per capita. The Powering Queensland Plan, despite the existence of huge coal resources, confirmed “the Government’s commitment to a 50% renewable energy target by 2030.” ACT emerged as the most progressive on links between climate change and energy use. SA was also very strong on renewables.^35^ Victoria’s Renewable Energy Roadmap recognised that its current electricity supply was unsustainable in terms of greenhouse gas emissions, at the time of publication 84% of its electricity came from brown coal and only 11% from renewable resources. This changed to 27% renewables and 68% brown coal in 2020.

 Health benefits of a change were rarely mentioned explicitly despite the evidence that reducing the use of fossil fuels would improve the health of those directly exposed to its pollution and the whole population’s health would benefit from moves to mitigate climate change.^2,36^ The NSW Renewable Energy Action Plan focused on attracting investment in renewable energy, building community support and attracting expertise in renewable energy. The plan does not detail any health or community benefits other than economic ones. The NSW Gas Plan recognised that there was significant community concern about using coal seam gas supplies because of their potential impact on human health and the environment. A review was commissioned to focus on the human health and environmental impacts of coal seam gas. IT gave cautious support for coal seam gas extraction if there were sufficient regulatory safe guards but also noted that there “*could be there could be unexpected events, learnings, or even accidents*” (p. iv)^37^ In response to these concerns the Plan noted:

 “*These benefits will be delivered by a strong, certain and trusted regulatory system, supported by science and information.”*

 The NSW Climate Change Fund Strategic Plan (2017-2022) stood out as making reference to health impact throughout the draft. Examples are:

 “*A stated policy direction was “Reduce climate change impacts on health and wellbeing” *(p. 8).

 “*…reduce the impacts of climate change on health and wellbeing, particularly in vulnerable communities, and manage the impacts of climate change on natural resources, ecosystems and communities” *(p. 13).

 “*…will reduce emissions and air pollution, improve public health, and make New South Wales more competitive” *(p. 21).

 “*Our efforts will also target carbon abatement projects that deliver health or biodiversity co-benefits” *(p. 22).

 “*Energy efficiency means lower costs, lower emissions, greater community wellbeing and a healthier economy*”(p. 23).

 Noted that more use of renewable energy and energy efficiency would “*free up valuable funds for frontline services such as education and health*” (p. 23).

 The ACT was also explicit about the advantages of dealing with climate change. Its ACT Climate Change Adaptation Strategy Living with a Warming Climate July 2016 states:

 “*By considering the future climate when making these decisions we will be in a better position to deal with the unavoidable impacts of climate change”* (p. 19).

 The ACT’s Renewable Energy Industry Development Strategy was explicit about developing “a vibrant, export-oriented renewable energy industry in the ACT.” Renewables are generally argued for on basis of ensuring new avenues for energy production and energy security and providing jobs in the industry – within this though, ensuring good quality of life and climate change impacts are discussed as secondary goals in SA (A Renewable Energy Plan for SA), ACT (ACT Sustainable Energy Policy) and Queensland in its Renewable Energy Plan.

 The Federal, WA and NT governments emerged as the jurisdictions still advocating the use of non-renewable sources rather than a transition to renewables, and explicitly justifying this on economic grounds. The National Energy Productivity Plan (p. 9) stressed the economic benefits of energy. For example, it says “*Energy productivity is about how much value we get from our investment in energy*.” The policy does mention reducing emissions, but that consideration is secondary to improving competitiveness and “growing the economy.” Under the Gillard Australian Labor Party government, the Australian Renewable Energy Agency was established in 2012 and has lasted the change of subsequent five Prime Ministers showing some Federal commitment to renewable energy, even though both major parties were committed to continuing the use of fossil fuels. The carbon tax introduced by the Gillard Government was repealed in 2014^15^ by an incoming conservative government. The Council of Australian Government produced a National Energy Productivity Plan which focused on “Boosting competitiveness, managing costs and reducing emissions.” The main preoccupation of the document was on reducing costs through improved efficiency which reflects the political imperative to do this. This document also made the statement that “*Energy productivity is a smart way to tackle climate change because it encourages economic growth while reducing emissions*” (p. 11). The WA policies concentrated on attractive business investment in their resources industry and very little attention was paid to health or sustainability. Thus, the Energy 2031 document made no reference to health or climate change although it did discuss making energy “cleaner.” Climate change is generally not located in a global or national context (such as international agreements on reducing carbon) within the policies but rather consideration of the local impacts of climate change to the jurisdiction in which each policy is located rather than considering global benefits.

###  Cost of Energy

**Figure 4:**
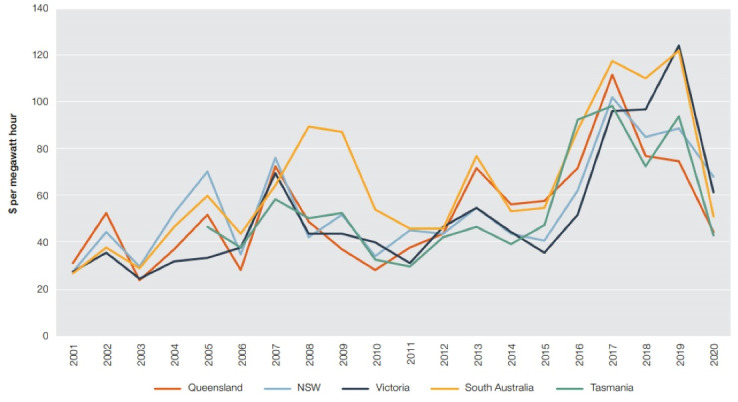
Wholesale Electricity Prices (2001-2020).^38^ Abbreviation: NSW, New South Wales.

 The price of energy in Australia rose significantly above inflation from 2007 (see Figure 4), making affordability a crucial issue.

 This trend was one social determinant of health that was widely recognized in the policies. The Tasmanian* Energy Strategy* (p. 1) was typical in noting:

 “*Our energy sector must deliver the lowest possible power prices that are genuinely sustainable over the longer term.”*

 Since the establishment of the market and privatisation in the 1990s and early 2000s, the inflation adjusted retail price for electricity has risen significantly in all states, which is contrary to the anticipated outcomes of competition reform.^32^

 Most commonly the policies framed consumers as being in a position to make rational choices and adopt a consumer choice framing. The National Energy Productivity Work Plan (p. 4) lists one of its strategies as “*Make choice easier. In order to make consumer choices easier and familiar to consumers and to promote consumer action to better manage their energy costs*.” The NSW Energy Efficient Action Plan referred to the rental paradox whereby the financial cost of energy inefficient dwellings is borne by the renter rather than the owner and so there was little incentive to make the dwelling more energy efficient.

 The ACT Sustainable Energy Policy (p. 15) maintained that market forces were the best way to control energy costs but recognised “*that there is a clear role to assist those in financial difficulty*.” Their action is to provide concessions on energy costs, making public housing more energy efficient and using education and retrofit measures to improve energy efficiency. The Tasmanian Strategy Plan dealt with energy costs through the welfare system and the provision of subsidy/discount strategies for low-income earners and recipients of welfare benefits to increase energy affordability.

 The policies we reviewed paid attention to some aspects of equity. “People before profits” (in the words of the SA Premier in the SA Our Energy Plan, p. 1) is a strong sentiment in SA, ACT and Tasmanian policy reflecting concern and resistance to negative implications that may stem from privatisation. SA, ACT, NSW, and Victoria all mention need to maintain low/affordable power prices for all to ensure access to energy and “quality of life.” However, it is seldom expanded to draw out what is meant specifically. NSW noted the existence of energy poverty (when households spend more than 10% of their income on energy) and introduced schemes to support low-income households. The NSW Climate Change Fund Plan also noted that they could “reduce the energy bills and improve the health and living standards for those most vulnerable in our society.” Many policies also recognised the fact that low-income households are the least able to purchase low energy appliances or have low-energy use homes. The SA Our Energy Plan noted a scheme to fit solar panels to publicly owned houses to enable low-income people to have access to energy efficiency and reduce their costs but has not extended this scheme to private sector tenants, where many low-income people live.

 We found very few references to Aboriginal and Torres Strait Islanders. When they are mentioned, they are generally referred to as a vulnerable consumer group or are mentioned in association with supply in rural communities. Land/country connection which are vital to Aboriginal and Torres Strait Islander wellbeing are not emphasized, except about this being a necessary consideration when determining mining sites. The NT stressed the important of a social licence for its Oil and Gas Guiding principles but only requested industry to act at an “acceptable level” in this regard. No special mention was made of relationships with traditional owners of the land and the focus in the policies was on the economic benefits. While Aboriginal Lands councils give recommendations about exploration on Indigenous land, the Minister has final say and grants permits for exploration of Indigenous land.

 Intergenerational equity is a driver for renewable energy policy- to reduce reliance on non-sustainable fuel sources so that quality of life can be ensured for future generations. The SA Our Energy Plan (p. 8) said “*Our children and grandchildren will inherit a cleaner and greener South Australia*.” The Victorian Department of Economic Development, Jobs, Transport and Resources (p. 27) set as an indicator:

 “*Improving the sustainable use of natural resources to ensure that future generations can continue to use Victoria’s resources to raise their quality of life.*”

 Victoria also refers to environment justice:

 “*The Sustainability Victoria Act includes principles of environmental justice. By environmental justice we mean decision-making processes that effectively integrate both long-term and short-term economic, environmental, social and equity consideration” *(Towards Sustainable Victoria, p. 10).

###  Interactions Between the Energy Sector and Other Social and Commercial Determinants of Health

 Some of the impact of the energy sector on health also comes from its interactions with a range of other social determinants. In terms of the built environment and housing strategies included targeted initiatives to improve the energy efficiency of aspects of the built environment, including schools (eg, in SA Our Energy Plan energy efficient lighting and solar panel installation) and private homes (mainly via rebates for solar panels for home owners and rating systems/education for home appliances), and some consideration of public housing (such as installation of energy efficient lights and solar panels in SA). The ACT Sustainable Energy Policy was the first jurisdiction to introduce strong energy efficiency standards for all new buildings. Its corporate plan of the Department of Environment and Planning was the outstanding example of integrated plans for urban development with increasing sustainable energy. We also found strategies targeted at reducing energy costs associated with running businesses via structural initiatives and/or discounts. This was best illustrated in Victoria where multiply sectors were integrated into the Department of Economic Development, Jobs, Transport and Resources so the overlapping concerns were addressed. The Tasmanian Renewable Energy Plan also noted that $ 6.25 million was committed to the Energy on Farms Policy. Health and well-being were however, not explicitly mentioned in either the ACT or Victorian plans.

 A further social determinant is the extent to which the energy sector provides employment opportunities. All the policies in states with extensive fossil fuel extractions (WA, Queensland, NSW, NT, Victoria) emphasized the need to preserve jobs in mining and used as this as a justification for continuing use of coal-based energy sources. A gradual transition to renewable energy was identified as a potential source of new energy sectors jobs by all jurisdictions. For example the Powering Queensland policy noted that its northern Queensland plan would “unlock around 2000 megawatts of renewable energy projects and support up to 4600 jobs.” Climate change was recognized in the ACT documents as a threat to the sustainability of health services if demand increases during heat waves and blackouts. Providing a reliable supply of and comparatively cheaper energy was viewed by all states as a means of attracting new businesses and so providing jobs. The Tasmanian strategy (p. 3) noted that competitive pricing would mean Tasmania would *“be better placed to attract investment in new industries*.”

 Transport was a frequently mentioned as a heavy user of energy resources. In the ACT, NSW, Queensland, Victoria and SA improving vehicle efficiency and encouraging the use of alternative fuels to reduce emissions and offset climate change was part of policy. The ACT’s Sustainable Energy Policy, for example committed to providing electric vehicle infrastructure and to make all its own fleet electric. The provision of low carbon use transport was also identified as an important aspect of quality of life in some policies. The ACT policy (p. 20) noted they would provide the infrastructure to “*make the sustainable and healthy travel options the easy choices.*”

## Discussion

 Despite the evidence that energy is a crucial social and commercial determinant of health which affects health in many ways our empirical study of energy policy in Australia indicates that health and wellbeing are only rarely explicitly considered in that policy. Figure 3 showing the conceptual framework we used to consider energy’s impact on health highlights two pathways to health impact: whole of population impacts largely through the extent to which energy is clean and sustainable and impacts on individuals such as cost which often result in significant inequities. We consider each type of health impact below.

###  Population-Wide Impacts

 The biggest risk to population health comes from the burning of fossil fuels. These fuels cause air pollution and dangerous climate change. Despite this massive threat to the health of all people on the planet Australia does not have a national energy plan designed to reduce the use of fossil fuels. The need to decarbonise energy sources in the interest of human health has been made strongly.^39,40^ Despite this two jurisdictions – the ACT and SA did have energy policies which addressed this issue and has seen the jurisdiction reduce reliance on fossil fuels and increase those on renewable energy. Thus, in a related study we assessed how SA had achieved the transition to renewable energy despite a generally unsupportive federal political environment^35^ and showed that a clear vision and support from public policy a renewable energy transition could be achieved in a privatised market. Significantly, this transition was instigated and subsequently enforced by a state government in a liberalised privately owned and operated market-based system in a nation with weak and inconsistent Federal government greenhouse gases reduction policies. By contrast the Federal government in the period covered by our review changed its policy dramatically as elections were won and lost and political leaders changed rapidly. The policies in all other jurisdiction did not substantially promote renewables.

 The main health risk in the Australian market is the intention to continue the use of fossil fuels as an important power source domestically and for export. Some of the Australian states have been proactive in this regard particularly SA and the ACT.

 Bacchi^41^ notes that silences in policy documents are as significant as the content. The most significant silence we noted was in the Federal government policies which gave very little attention to the impact of fossil fuels on the environment. There has been active promotion of gas as a ‘transition fuel,’ particularly by Coalition government in power until May 2022, and consequently active promotion of gas as being a part of a cleaner energy future and recently as part of the post coronavirus disease 2019 (COVID-19) economic recovery plan. This approach fails to recognise that gas is a fossil fuel that also produces greenhouse gas emissions. We also found no evidence that the Federal government’s policies to support a high carbon energy policy were influenced by health and environmental concerns. Yet the most significant area of energy policy likely to reduce human health is the continued use of fossil fuels.^3,42^ In 2018-2019, Australia’s domestic energy consumption was still largely dependent on fossil fuels (94%) and while the use of renewables was increasing, it only accounted for 6.4% of Australia’s total energy consumption.^43^ Spatial impacts arise because renewable energy use is not evenly distributed with ACT, SA, and Tasmania having higher renewable energy generation and use and other regions minimal renewable generation. Fossil fuels are highly subsidised by taxpayers,^44^ despite strong evidence that renewable sources of energy offer significant advantages.^1^ They do not irreversibly deplete finite resources, and most have a lower climate footprint than fossil fuels. They have the potential to pose minimal health risks and can yield social and economic co-benefits. However, all energy sources have some health and environmental impacts. Issues of land use, maintenance, materials inputs, and energy storage raise concerns about environmental, occupational, and community health impacts. For instance, concern has been raised concerning the impact of new mining activity to produce minerals for renewable energy sources.^45^

 Australia is not only a major user of fossil fuels it is also a major exporter. Coal alone accounted for 15% ($60 billion) of the country’s export income in 2018 and gas a further 8% ($38 billion).^20^ These export industries are predominantly situated in three states. Coal exports from Queensland and NSW and gas from WA. This economic pre-eminence has given the fossil fuel lobby significant political influence over some state and federal governments^16,17,21^ and Australian energy policy shows only scant regard for health and wellbeing.

 Our framework (Figure 3) indicates links between energy policies and mental health which have been previously documented.^46^ People are concerned about how climate change causes rises in sea levels, more frequent and intense bushfires, and sadness about the degree of biodiversity loss and each of these can impact on mental health. Especially for First Nation Australians connection to Country is threatened by coal mining activity and gas extraction.^47^ Mental health is also affected by the stress caused by high energy bills and the inability to afford adequate energy for cooling and heating for low-income households.^48^ Yet these considerations were not found in our policy review.

 The policy environment for energy shapes the ways in which ownership and the nature of the energy market position it as an important commercial determinant of health. The decisions made in privatised energy markets will reduce population health equity in the longer term through pathways that include the extent of greenhouse gas, other damaging emissions, and price. These pathways determine the sustainability and greenness of energy supply, whether access to energy is equitable and reliable and whether there are hazards to people or the environment from its generation. The Australia Institute’s analysis of the privatisation of electricity in Australia notes it has led to fragmentation, duplication, and waste.^49^ The privatisation of energy in Australia resulted in a rapid price rise and concern about price is clear in all the policy documents. The increase in price came to be a major political issue and contributed to the uptake of solar panels by individual homeowners so in terms of reducing carbon pollution could be seen to be of benefit and demonstrates a commitment to reducing emissions. However, in terms of equity only those who could afford the solar benefits benefited most and low-income people did not have the resources.^50^ One aspect of price that received very little consideration in Australian policies was strategies to enable people to use less energy by enforcing building regulations which specify a certain standard of energy efficiency through, for example, insulation and good housing design. We note that improved energy efficiency standards for housing is vital because the critical contribution of improvements in building energy efficiency to minimising energy demand growth and resultant emissions has been recognized by the International Energy Agency.^51^ Thermally efficient housing will both improve health outcomes and reduces household energy costs over time. However, the modest upfront cost of much improved housing is one property developers and landlords are reluctant to make despite the much more substantial medium- and longer-term benefits. Mandating housing efficiency standards is a policy change that would have substantial health, equity and affordability benefits.

 A further silence in energy policy is the need for clean air to reduce the impacts of pollution. The burning of fossil fuels in the internal combustion engines of motor vehicles and for electricity generation produces localised pollution containing a cocktail of noxious chemicals and particulates.^52,53^ These pollutants elevate and exacerbate cardiovascular and respiratory risks, complaints such as diabetes and dementia and contribute to adverse birth outcomes such as low weight and prematurity, while some chemical pollutants such as benzene are known carcinogens.^52,54-59^ It is estimated outdoor air pollution causes between 2400 and 3000 premature deaths in Australia each year, not including cancers, and costs up to $17.8 billion.^60^ Despite these drawbacks gasoline and patrol are heavily subsidised in many countries including Australia.^61^

 In Australia, exposure to brown pollution from motor vehicles is also spread inequitably because it is concentrated within 150 metres of congested or high-volume roads. Therefore, adverse health effects from it are disproportionally felt by those who live, work, study, or spend long periods travelling in such locations.^52,54,62^ Similarly, the risk from premature death or sickness from coal pollution is estimated to be 3 to 4 times higher for people living within 50 km of a coal generator.^63,64^

###  Limitations

 This study examined Australian energy policy at a particular point in time and was concerned with its impact on health but our qualitative analysis did not extend to studying implementation. Nonetheless our analysis of all strategic policy documents does enable us to make a considered assessment of how healthy Australian energy policy is and what a healthy energy system would look like.

## Conclusion and Policy Implications

 Our analysis offers pointers for health promoting intersectoral projects such as Healthy Cities^65^ and Health in All Policies.^66^ Part of the consideration of these initiatives should be consideration of how energy sources, prices and availability affect health. They can also play an important role in advocating for cleaner and healthier energy. In the past decade the energy landscape in Australia has changed significantly in response to local political and global changes but through most of this time has not had a coherent consistent plan for transitioning to a zero-carbon nation. Our study indicates the need for a national plan for the energy and the transition to no-carbon energy and mechanisms to link the nine jurisdictions. These needs have also been recognised by other policy analysis.^67^ Australia does not have an overarching energy plan which meant there was no Federal government commitment to develop policy that would set a path to a reduction of the use of fossil fuels and transition to renewable energy. Had health been a major consideration in the development of energy policy then this transition may have occurred in the period we studied. Drawing on the pathways identified in Figure 3 and our analysis of policy we identify the following seven dimensions of a healthy energy policy which could be the basis for all countries with adaption to local contexts.

1. Development of an over-arching national energy plan which considers the health impact of energy.
2. Explicit consideration of the health impact of energy systems in all energy policies.
3. Policy commitment to achieve the carbon target of net zero by 2050 and by 75% by 2030.
4. Ensuring retraining for workers who lose jobs as fossil fuels are phased out and support for affected communities.
5. Energy production, distribution and retail in public ownership or under effective public sector regulation.
6. Top-rated thermal efficiency in housing incentivised with compulsory standards for new constructions and retrofitting existing building including grants for low-income earners, tax incentives for landlords and doing so for all public housing.
7. Health sector exercises its stewardship responsibilities by advocating for healthy energy policy and conducting health impact assessments on new energy initiatives.

Adoption of the policies outlined above will require advocacy from health departments, public health professional associations and citizen groups. Currently in Australia the strongest energy lobbying voice^68,69^ comes from the fossil fuels industry which makes substantial donations to political parties in order to gain access to both governments and oppositions with a view to influencing policy.^70^ This is one of the likely reasons that Australian energy policy still favours fossil fuels.

 An important step in reducing the adverse impact of energy policy on health is likely to be the conduct of health impact assessments on aspects of energy.^71^ A number of examples are available from the WHO.^10^ Perhaps most importantly, policy should force the energy sector to accept that because its activities determine population health and health equity it has a responsibility to consider health outcomes and their long-term costs. To this end the Director General of WHO has convened a High-Level Coalition on Energy and Health^72^ to increase co-operation between the sectors. A similar coalition would be helpful within countries.

 Energy is vital to our lives yet has rarely been assessed in terms of its health impacts in recent Australian policies. There are multiple ways in which energy influences health and understanding these and then mitigating them through policy is an increasingly important task especially given the climate and environmental emergency the world is facing.

## Acknowledgement

 This research was funded by ARC grant DP160100244. We thank Toni Delany-Crowe for her contribution to the initial analysis for this paper.

## Ethical issues

 No ethics approval was required as the research reports on an empirical study of policy documents.

## Competing interests

 Authors declare that they have no competing interests.

## Authors’ contributions

 FB wrote the original draft. MM, CM, and MH provided critical feedback on each draft. FB and CM won the funding. FB, MM, and CM analysed the policies. FB supervised the research.
